# Prevalence of burnout among intensive care physicians: a systematic review

**DOI:** 10.5935/0103-507X.20200076

**Published:** 2020

**Authors:** Filippo Sanfilippo, Gaetano Joseph Palumbo, Alberto Noto, Salvatore Pennisi, Mirko Minieri, Francesco Vasile, Veronica Dezio, Diana Busalacchi, Paolo Murabito, Marinella Astuto

**Affiliations:** 1 Department of Anesthesia and Intensive Care, A.O.U. “Policlinico-Vittorio Emanuele” - Catania, Itália.; 2 School of Anaesthesia and Intensive Care, University Hospital “G. Rodolico”, University of Catania - Catania, Italy.; 3 Department of Human Pathology of the Adult and Evolutive Age “Gaetano Barresi”, Division of Anesthesia and Critical Care, University of Messina - Messina, Italy.; 4 Department of General Surgery and Medical- Surgical Specialties, Section of Anesthesia and Intensive Care, University of Catania - Catania, Italy.

**Keywords:** Burnout, professional/epidemiology, Working conditions, Physicians/psychology, Occupational diseases/epidemiology, Depression, Prevalence, Intensive care units, Esgotamento profissional/epidemiologia, Condições de trabalho, Médicos/psicologia, Doenças ocupacionais/epidemiologia, Depressão, Prevalência, Unidades de terapia intensiva

## Abstract

**Objective:**

We performed a systematic review to summarize the knowledge regarding the prevalence of burnout among intensive care unit physicians.

**Methods:**

We conducted a systematic review of the MEDLINE and PubMed® databases (last update 04.02.2019) with the goal of summarizing the evidence on burnout among intensive care unit physicians. We included all studies reporting burnout in intensive care unit personnel according to the Maslach Burnout Inventory questionnaire and then screened studies for data on burnout among intensive care unit physician specifically.

**Results:**

We found 31 studies describing burnout in intensive care unit staff and including different healthcare profiles. Among these, 5 studies focused on physicians only, and 12 others investigated burnout in mixed intensive care unit personnel but provided separate data on physicians. The prevalence of burnout varied greatly across studies (range 18% - 49%), but several methodological discrepancies, among them cut-off criteria for defining burnout and variability in the Likert scale, precluded a meaningful pooled analysis.

**Conclusion:**

The prevalence of burnout syndrome among intensive care unit physicians is relatively high, but significant methodological heterogeneities warrant caution being used in interpreting our results. The lower reported levels of burnout seem higher than those found in studies investigating mixed intensive care unit personnel. There is an urgent need for consensus recommending a consistent use of the Maslach Burnout Inventory test to screen burnout, in order to provide precise figures on burnout in intensive care unit physicians.

## Abstract (pt)

**Objetivo:**

Realizar uma revisão sistemática para sumarizar o conhecimento relativo à prevalência de burnout entre médicos atuantes na unidade de terapia intensiva.

**Métodos:**

Conduzimos uma revisão sistemática nas bases de dados MEDLINE e PubMed® (última atualização em 4 de fevereiro de 2019), com o objetivo de resumir a evidência a respeito de burnout entre médicos atuantes em unidades de terapia intensiva. Incluímos todos os estudos que relatavam burnout em trabalhadores na unidade de terapia intensiva, segundo o Inventário de Burnout de Maslach e, a seguir, triamos os estudos quanto a dados relativos a burnout especificamente em médicos atuantes na unidade de terapia intensiva.

**Resultados:**

Encontramos 31 estudos que descreviam burnout em membros da equipe da unidade de terapia intensiva e incluíam diferentes perfis de profissionais de saúde. Dentre estes, cinco estudos se focalizavam apenas em médicos, e 12 outros investigavam burnout em uma mescla de profissionais atuantes na unidade de terapia intensiva, mas forneciam dados à parte relativos aos médicos. A prevalência de burnout teve grande variação entre os estudos (variando entre 18% e 49%), porém diversas discrepâncias metodológicas, dentre elas os critérios de corte para definição de burnout e variabilidade da escala de Likert, impediram uma análise agrupada significativa.

**Conclusão:**

A prevalência da síndrome de burnout entre médicos atuantes na unidade de terapia intensiva é relativamente alta, porém heterogeneidades metodológicas significantes exigem precauções na interpretação de nossos resultados. Os níveis mais baixos de burnout relatados parecem mais elevados do que os identificados em estudos que investigaram uma mescla de profissionais da unidade de terapia intensiva. Há uma necessidade urgente de consenso que recomende o uso consistente do Inventário de Burnout de Maslach para triar a presença de burnout a fim de fornecer dados precisos a respeito de burnout entre médicos atuantes na unidade de terapia intensiva.

## INTRODUCTION

Psychological stress among medical disciplines is a “hot-topic”, and several specialties are deemed at high risk.^(1)^ When faced properly with an adequate cognitive approach and coping strategies, stress can exert beneficial effects.^(2)^ Indeed, the ability to tackle challenging scenarios may build self-confidence and enhance the sense of well-being and of being helpful.^(3)^ However, an exaggerated degree of stress and/or a suboptimal approach to stressful situations may lead to decreased satisfaction, undermining a physician’s mental and physical health,^(4-7)^ ultimately increasing the risk of developing a psychological syndrome known as burnout.^(8)^

In the 11^th^ Revision of the International Classification of Diseases, burnout is classified as an occupational phenomenon but not a medical condition.^(9)^ Burnout is described as a syndrome resulting from chronic workplace stress that has not been successfully managed, and it is characterized by three dimensions (main components): high emotional exhaustion (EE), high depersonalization (DP) and low personal accomplishment (PA).^(10)^ In brief, EE is a subjective work-related sense of fatigue (feelings of energy depletion or exhaustion), DP is a defense mechanism in the attempt to separate oneself from work (feelings of negativism or cynicism work-related), and low PA represents a feeling of frustration with work-related achievements (reduced professional efficiency). Burnout differs from depression because it is related to the work environment. It develops in response to chronic interpersonal stressors,^(11)^ and it is more likely to occur in the absence of appropriate support from healthcare organizations.^(12-14)^ Its presence negatively affects patients’ care^(15-17)^ and physicians’ professionalism;^(18-21)^ moreover, it has been associated with relationship impairment among team members,^(22)^ decreased work activity,^(23)^ worsened quality of care delivered,^(19,24,25)^ and higher healthcare costs.^(26)^ Although work-related, burnout seems to play a role in the development of major depression or substance abuse.^(27)^ Therefore, it is easy to understand why physician burnout seriously affects healthcare professionals’ performances and well-being, and the implementation of strategies to reduce its impact is under scrutiny.^(28)^

Burnout is reaching epidemic levels among physicians, with prevalences in several disciplines reported to be over 50%, and those working in the intensive care unit (ICU) have been reported to have the highest prevalence of burnout.^(29)^ This finding is not entirely surprising since the ICU is certainly one of the most stressful environments, continuously exposing physicians to great responsibilities and stressful situations, such as the management of life-threatening scenarios, decisions to withdraw life-supporting strategies, and dealing with multiple and difficult tasks simultaneously. Moreover, the work pattern is certainly more stressful than that of other medical disciplines, including overnight duties and shifts during weekends and festivities.

Several studies and surveys have studied the prevalence of burnout in the ICU setting. One study reported that the prevalence of burnout in the ICU varies from 0% to 70%,^(30)^ while another found a narrower (though still large) range (6% - 47%).^(31)^ Nonetheless, there was gross heterogeneity in their design: different ICU healthcare professionals were included (physicians, residents, nurses, physiotherapists), and different countries and regions and different ICU settings (general, neuro, cardiac) were examined. In consideration of such heterogeneity and considering that a systematic assessment of physicians only has not yet been conducted, we performed a systematic review to summarize the knowledge regarding the prevalence of burnout in ICU physicians.

## METHODS

We undertook a systematic, web-based, advanced literature search, using the National Health Service (NHS) Library Evidence tool, on the prevalence of burnout in ICUs. We followed the approach suggested by the Preferred Reporting Items for Systematic Reviews and Meta-Analyses (PRISMA) statement for reporting systematic reviews.^(32)^ However, since the search was already performed, registration on PROSPERO was not possible.

### Systematic search

To identify relevant articles, an initial computerized search of MEDLINE and PubMed® was conducted from inception until 2 October 2018; with the findings from this search, we started the data extraction. The search was then updated on 4 February 2019, limiting the search to the end of 2018. The manuscript was amended with the new findings.

Our core search was structured on the combination of two groups of terms. The first group included only the term “burnout,” while the second group included the following words: “intensive care” and “critical care”. Comparable search strategies have been adopted by similar studies.^(30,31,33)^

### Study eligibility, data extraction and outcomes

Inclusion criteria were pre-specified according to the PICOS approach (Table 1). Study selection and determination of eligibility for inclusion in the systematic review and subsequent data extraction were performed independently by five reviewers with cross-checking (two assessors for each article). The included articles and extracted data were subsequently reviewed by the other two authors. Each discrepancy was discussed with the initial assessors. Discordances were resolved by involving the senior author.

**Table 1:** PICOS approach for selecting studies in the systematic search

PICOS	Characteristics of studies included in the systematic search
Participants	Intensive care physicians
Intervention	Assessment of burnout syndrome with any form of the MBI questionnaire
Comparison	None
Outcomes	Risk of burnout syndrome evaluated either overall or according to subscales for burnout
Study design	Prospective surveys including at least 10 intensive care physicians

MBI - Maslach Burnout Inventory

Language and timing restrictions were applied: we read the full manuscript only for articles published in English or Italian, and we limited our search to the period of 1999-2018 (i.e., the last 20 years). A manual search was conducted independently by three authors and included exploration of the lists of references from the studies found in the systematic search. We excluded book chapters, reviews, editorials and letters to the editor for the qualitative synthesis. We expected high heterogeneity for data concerning burnout, partly because of the different tools used for assessing burnout. Since the most commonly used burnout assessment tool is the Maslach Burnout Inventory (MBI), in order to facilitate the aggregation and comparison of data, we decided to include only studies assessing burnout using a version of the MBI. The aim of this systematic search was to summarize knowledge and provide broader insight into the topic.

## RESULTS

**Figure 1:**
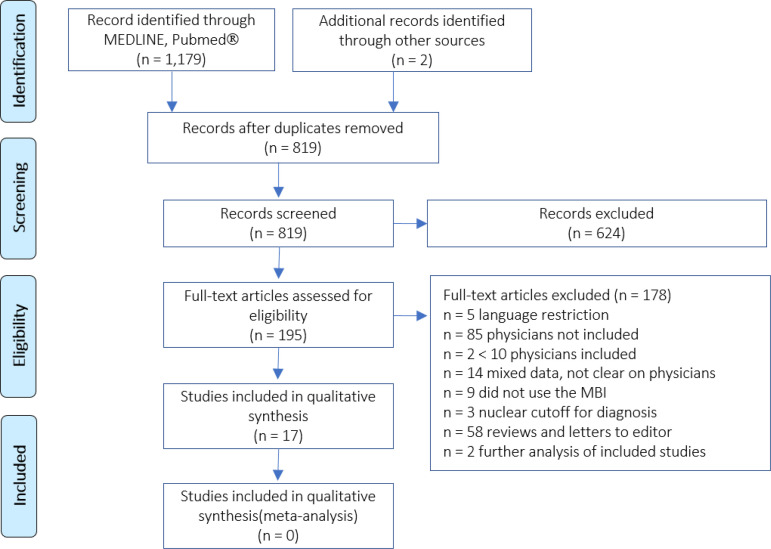
Preferred Reporting Items for Systematic Reviews and Meta-Analyses (PRISMA) flowchart of the conducted screening. MBI - Maslach Burnout Inventory. Adapted from: Moher D, Liberati A, Tetzlaff J, Altman DG; PRISMA Group. Preferred reporting items for systematic reviews and meta-analyses: the PRISMA statement. PLoS Med. 2009;6(7):e1000097.

The literature web-based search yielded a total of 754 citations on PubMed^®^ and 425 on MEDLINE. The manual search identified two other articles. After the exclusion of 362 duplicates, 819 records were screened, but only 195 assessed the topic of burnout in intensive care. Of these, we excluded 178 articles after assessment for eligibility; therefore, we included in our literature summary a total of 17 articles (Figure 1). Of these, we found that five studies directly assessed burnout in ICU physicians only (n = 5/17, 29%), while the other twelve studies (n = 12/17, 71%) included physicians as well as other professionals. In particular, during our screening of full texts, 26 studies were potentially eligible for inclusion in the qualitative synthesis as they involved surveys on burnout in mixed ICU personnel (nurses and/or physiotherapists and/or auxiliary staff). However, from the full texts of these mixed studies, we were able to retrieve separate data on physician burnout in almost half (n = 12/26, 46%), which allowed us to increase the pool of studies for our qualitative synthesis by over three times. We attempted to further increase the amount of data by emailing the corresponding authors of the remaining 14 studies, but unfortunately, we did not get any responses in two attempts (the second email sent two weeks after the first).

Table 2 summarizes the results of the included studies along with the response rate (for physicians, if provided, or overall, if not, in case of mixed studies), ICU setting and country/region, the type of MBI questionnaire used, the finding on burnout and/or on its domains (EE, DP, PA), and the criteria used for burnout diagnosis. The reported response rate varied greatly (range: 30% to 90%), and the data on physician participation in the study were not always available. Similarly, the presence of severe (or high degree) burnout varied and was reported in the range of 18% to 49%. The vast majority of studies (n = 15/17) investigated burnout by means of the full version of the MBI questionnaire (22 questions; EE = 9, DP = 5, PA = 8); one used an abbreviated MBI (9 questions),^(34)^ and another used an almost full version (21 questions) coupled with four questions on “consternation”.^(35)^ The cutoffs for diagnosis of burnout varied greatly, with EE ranging from 24 to 31, DP from 9 to 13 and PA from 29 to 33. Moreover, the interpretation of these cutoffs was even more cumbersome because the Likert scale used for the MBI varied (scales ranging from 4 to 7 points), there was an unclear range in 3 studies (18%), and the cutoff was not specified at all in 7/17 (41%) studies.

**Table 2:** Included studies reporting data on physician burnout in the intensive care unit

Authors	Population and response rat e	ICU setting, country/region	Burnout questionnaire Likert scale	Burnout in physicians	Subscale scores for physicians	Criteria for burnout classification
Colville et al.^(34)^	74 physicians (and 285 nurses) RR 51% overall, not available for categories	Seven ICUs at two hospitals (pediatric, cardiac, neuro, general) United Kingdom	Abbreviated MBI 9q Likert not specified	High 49%	Not reported	EE (≥ 27 points), DP (≥ 10 points) and PA (≤ 33 points), calculated as weighted/prorated scores Burnout if high levels of EE or DP
Lederer et al.^(35)^	33 physicians (and 150 nurses) answered RR 59% overall	5 ICUs Innsbrusk, Austria	Modified MBI (25q: EE 9, DP 5, PA 7, and Consternation 4) 6-point scale (1 to 6)	High in 45% (15/33)	Not reported	High risk burnout defined as EE mean value ≥ 4 or DP mean value ≥ 4, or PA mean value ≤ 4.
See et al.^(36)^	992 physician (out of 1296) and 3100 nurses (out of 4895, RR 63.3%) RR of 76.5% physicians	Medical, surgical and mixed ICUs Asia	Full MBI (22q: EE 9, DP 5, PA 8) Likert not specified	50.3%	EE high: 39.9% DP high: 35.2% PA low: 54.7%	EE (≥ 28 points), DP (≥ 11 points) and PA (≤ 33 points) Overall high burnout if high score in EE or DP domain
Barbosa et al.^(37)^	67 physicians RR not available	Various types of ICUs (general, cardiac, pediatrics/neonatal, others) Brazil	Full MBI (22q: EE 9, DP 5, PA 8) 5-point scale (1 to 5)	High in at least 1 domain 70.1% High in all domains 17.9%	EE high:41.8% DP high:37.3% PA low: 58.2%	EE (≥ 27 points), DP (≥ 10 points) and PA (≤ 33 points) Burnout diagnosed if at least one domain altered
Fumis et al.^(38)^	283 ICU providers, 33 (out of 49) physicians, with a RR of 67.3%	General and neuro ICUs + step-down ICU Brazil	Full MBI (22q: EE 9, DP 5, PA 8) Likert not specified	Severe 18.2%	EE 27.3%	EE (≥ 27 points), DP (≥ 10 points) and PA (≤ 33 points) Severe if all domains altered
Garcia et al.^(39)^	35 physicians (compared with 35 general pediatricians) RR 90% overall	2 pediatric ICUs Brazil	Full MBI (22q: EE 9, DP 5, PA 8) 7-point scale (0 to 6)	High in 1: 71% High in 2: 51% High in 3: 17%	EE high: 63% DP high: 40% PA low: 29%	EE (≥ 27 points), DP (≥ 13 points) and PA (≤ 30 points)
Tironi et al.^(40)^	180 (out of 600 randomly selected) physicians RR 30% physicians	60 ICUs (of responding physicians 70.6% worked in adult ICUs, 29.4% in pediatric/neonatal ICUs) Brazil	Full MBI (22q: EE 9, DP 5, PA 8) 7-point scale (0 to 6)	Overall High in 1: 36.7% High in 2: 20.0% High in 3: 38.3% Adult High in 1: 33.1% High in 2: 23.6% High in 3: 36.2% PED/Neonatal High in 1: 45.3% High in 2: 11.3% High in 3: 43.4%	Overall EE high: 50.6% DP high: 26.1% PA low: 15.0% Adult EE high: 51.9% DP high: 30.7% PA low: 18.9% PED/Neonatal EE high: 47.2% DP high: 15.1% PA low: 5.7%	EE (≥ 27 points), DP (≥ 13 points) and PA (≤ 31 points)
Embriaco et al.^(41)^	978 physicians (62% attending physicians, 14% fellows, 24% interns and residents) RR 82.3%	189 of 318 various ICUs (59.4%) France	Full MBI (22q: EE 9, DP 5, PA 8) 7-point scale (range unspecified)	High 46.5% Moderate 30.2% Low 23.3%	Overall: EE high: 19% DP high: 37% PA low: 39%	EE (≥ 27 points), DP (≥ 10 points) and PA (≤ 33 points) High if global MBI score ≥ -8, moderate level -21 to - 9, low level -45 to -22
Garrouste-Orgeas et al.^(42)^	330 physicians (out of 401) (and 1204/1587 nurses) RR 82% physicians	31 mixed, surgical or medical ICUs France	Full MBI (22q: EE 9, DP 5, PA 8) 7-point scale (0 to 6)	Def 1: 2.5%Def 2: 40.3%	EE high: 10.6% DP high: 24.5% PA low: 31.5%	EE (≥ 30 points), DP (≥ 12 points) and PA (≤ 33 points) Definition 1 High if all domains altered Definition 2 High if global MBI score ≥ - 9
Malaquin et al.^(43)^	32 physicians (and 129 nurses or nursing assistants) RR 90% overall	3 ICUs at single center (medical-surgical, cardiothoracic and vascular, and neurosurgical) France	Full MBI (22q: EE 9, DP 5, PA 8) Likert not specified	56% moderate 0% high	EE severe: 6% EE moderate: 32% DP severe: 29% DP moderate: 29% PA severe: 48% PA moderate: 22%	Low, 1 domain altered Moderate, 2 domains altered Severe, all domains altered
Giannini et al.^(44)^	71 physicians and 127 nurses (at baseline) RR 89% overall	8 ICUs (7 mixed medical-surgical, 1 pediatric) Italy	Full MBI (22q: EE 9, DP 5, PA 8) Likert not specified	High 32.4%	Not reported	EE (≥ 24 points), DP (≥ 9 points) and PA (≤ 29 points) High if global MBI score ≥ -9
Raggio et al.^(45)^	Physicians (25) and nurses responding in total) RR not reported	1 mixed and 1 post-transplant ICU Italy	Full MBI (22q: EE 9, DP 5, PA 8) 7-point scale (0 to 6)	Not reported	EE high: 36% DP high: 56% PA high: 28%	EE (≥ 24 points), DP (≥ 9 points) and PA (≤ 29 points)
Shenoi et al.^(46)^	275 (out of 686) physicians (20 excluded for questions unanswered) RR physicians 40% (37% excluding the 20)	Pediatric ICU, non-random sample of physicians United States	Full MBI (22q: EE 9, DP 5, PA 8) Likert not specified	Severe 21.0%	EE high: 34% DP high: 21% PA low: 20%	EE (≥ 27 points), DP (≥ 10 points) and PA (≤ 32 points) Severe burnout defined as high EE associated with high DP or low PA scores
Ntantana et al.^(47)^	149 (out of 221) physicians (and 320 nurses) RR physicians 67.4%	21 multidisciplinary ICUs > 6 beds Greece	Full MBI (22q: EE 9, DP 5, PA 8) Likert not specified	Not reported	EE high: 22.8% DP high: 42.3% PA low: 54.4%	EE (≥ 27 points), DP (≥ 10 points) and PA (≤ 33 points)
Teixeira et al.^(48)^	82 physicians and 218 nurses RR 78% physicians	10 adult ICUs (of 13 invited) Portugal	Full MBI (22q: EE 9, DP 5, PA 8) 7-point scale (range unspecified)	High: 24.7%	Not reported	EE (≥ 25 points), DP (≥ 10 points) and PA (≤ 32 points) High burnout if all domains altered
Merlani et al.^(49)^	Physicians (465 of 678), nurses and nurse's assistants RR 69% physicians	72 of 92 ICUs (RR 80%) Switzerland	Full MBI (22q: EE 9, DP 5, PA 8) 4-point scale (range unspecified)	High: 31%	Not reported	High if global MBI score ≥ -9
Galván et al.^(50)^	162 (out of 270) physicians RR 60% physicians	Pediatric ICU Argentina	Full MBI (22q: EE 9, DP 5, PA 8) Likert not specified	41%	EE high: 25% DP high: 19% PA low: 6%	EE (≥ 27 points), DP (≥ 10 points) and PA (≤ 32 points) Burnout high risk defined if at least one domain altered

ICU - intensive care unit; RR - response rate; MBI - Maslach Burnout Inventory; EE - emotional exhaustion; DP - depersonalization; PA - personal accomplishment.

From a geographical perspective, the largest (and more recent) study found in our search was a continental Asian survey containing data on 992 physicians with a high response rate (above 75%).^(36)^ Brazil and France had the greatest number of studies investigating burnout in ICU physicians (four^(37-40)^ and three^(41-43)^ publications, respectively), followed by Italy with two studies.^(44,45)^ The other seven studies included ICU physicians working in the United States,^(46)^ Austria,^(35)^ Greece,^(47)^ Portugal,^(48)^ United Kingdom,^(34)^ Switzerland^(49)^ and Argentina.^(50)^

Table 3 summarizes further findings retrieved from the included studies that were deemed of interest by the authors, with a focus on factors associated or correlated with burnout.

**Table 3:** Findings retrieved from the included studies and deemed of interest, with particular focus on factors associated or correlated with burnout

Study	Variables found to be associated or correlated with burnout
Colville et al.^(34)^	The analysis on the overall staff (physicians and nurses) showed that burnout significantly overlapped with post-traumatic stress disorder and anxiety. Multivariate analyses on variables correlated with burnout were performed according to two models. Resilience and being a doctor were the strongest predictors of reporting burnout. Attending a debriefing was correlated with halving the risk of burnout, whereas venting emotion and using alcohol were correlated with increased burnout reporting.
Lederer et al.^(35)^	The authors also included four questions in the MBI that were related to consternation, which is defined as fear resulting from the awareness of being susceptible to psychological trauma. This element was not included in the burnout definition and thus did not alter the results on burnout. The authors did not find significant differences in burnout between the subgroups for age, gender, level of training, years of employment or family status. ICU personnel with fully established burnout planned to change professions more frequently than participants with no burnout.
See et al.^(36)^	In the multivariate analysis of this Asian continental survey, protective factors against burnout for physicians were religiosity, years of experience in the current department, shift work and number of stay-at-home calls. The number of days worked per month was positively correlated with higher burnout.
Barbosa et al.^(37)^	The study found that 50% of the participants who did not practice physical activity had high levels of EE. Several sources of stress were investigated, but their influence on burnout was not directly evaluated.
Fumis et al.^(38)^	Moral distress (evaluated by the Moral Distress Scale-Revised questionnaire) correlated moderately with EE and weakly with PA (inversely) and DP.
Garcia et al.^(39)^	The authors found higher burnout among pediatric intensivists than among general pediatricians. No other demographics or personal characteristics were associated with burnout in the univariate analysis.
Tironi et al.^(40)^	Study including only physicians who were working in adult or pediatric/neonatal ICUs. Functional characteristics and occupational stress factors were reported, but their association with burnout was not analyzed. When considering high scores in all the three dimensions simultaneously, burnout was only observed in doctors working in adult ICUs (7.1%).
Embriaco et al.^(41)^	In this study, 50% of physicians with high levels of burnout wished to leave their job. The univariate analysis showed higher levels of burnout in females, in younger staff and in those not married and not having children. Burnout was also associated with withholding or withdrawing treatment, workload, and recent conflicts with nurses, families and colleagues. In the multivariate analysis, the factors remaining correlated with burnout were female gender, workload, and conflicts. Protective effects were good quality relationships with the chief nurse and nurses. The authors subsequently published other results of this study highlighting that, in the same cohort of physicians, depressive symptoms were correlated with high levels of burnout (Embriaco N et al. Annals of Intensive Care 2012).
Garrouste-Orgeas et al.^(42)^	Burnout was correlated with depression scores, as evaluated by the Centre of Epidemiologic Studies Depression scale, but not with safety attitudes, as evaluated by the Safety Attitude Questionnaire - ICU version. The study focused on the association between burnout and medical errors in the ICU.
Malaquin et al.^(43)^	Prevalence of burnout was not different between physicians and non-physicians or among the three different ICUs in this single center study. Severe burnout was more likely due to low PA than to high DP or EE. However, severe burnout was observed only in the cardiothoracic and vascular ICU (9%). After multivariate analysis, only the prevalence of depressive symptoms, low well-being and absence of a hobby were correlated with burnout.
Giannini et al.^(44)^	The study evaluated burnout and other outcomes, such as anxiety, in nurses and physicians regarding the liberalization of visiting times in the ICU. Staff was surveyed at three time-points, and nurses always had a significantly greater predominance of high burnout levels. Staff with favorable opinions regarding liberalization had lower burnout levels. Burnout level increased during the surveyed period in both nurses and physicians.
Raggio et al.^(45)^	The study evaluated the prediction of burnout according to results of the "profile of mood state" questionnaire that studies the profile of the state of mood in the previous week (58 specific sensations). Apart from the state of mood, the study showed a higher degree of DP in male physicians and a higher degree of EE in female physicians.
Shenoi et al.^(46)^	Approximately two thirds of the investigated population of physicians recently considered leaving their job in the pediatric ICU. Burnout and severe burnout were significantly associated with willingness to leave the job (4 and over 9 times higher risk, respectively). Severe burnout was significantly associated with psychological distress (over 8 times higher risk). The correlation between the EE score and the psychological distress score was moderate to high, while it was low to moderate for DP and PA.
Ntantana et al.^(47)^	In the overall study evaluating nurses and physicians, female sex was associated with higher EE and lower PA scores. Regarding EE, the multivariate analysis found a correlation with job satisfaction, satisfaction with end-of-life care, feelings of isolation after providing end-of-life care, neuroticism and extraversion traits.
Teixeira et al.^(48)^	Physicians and nurses were included in this study, and the data were mostly reported as pooled outcomes. The only significant difference found was the lower scores for EE in physicians compared with nurses (17 vs 20, respectively). The authors subsequently published other results for the same cohort highlighting that nurses' burnout (and in particular EE) was associated with ethical decisions (withdrawing or withholding treatments, terminal sedation), while this was not the case for physicians (Teixeira C et al J Med Ethics 2013).
Merlani et al.^(49)^	The study evaluated stress and burnout in a mixed population of physicians, nurses and nurse assistants in Swiss ICUs. The latter healthcare workers had significantly higher burnout (41%)than nurses (28%) and physicians (31%). The multivariate analysis in the overall population showed a higher risk of burnout according to individual factors (males, having no children, being younger than 40 years old), patients' related factors (higher ICU mortality), and organizational factors (working in German-speaking ICUs and having a lower proportion of females nurses). Moreover, a positive answer to the question about "Feeling stressed" was the predominant independent factor increasing burnout risk.
Galván et al.^(50)^	The score in the PA domain was independent from the scores in the EE and DP domains, while the latter scores had significant associations between them. In the multivariate analysis, being certified as a pediatric ICU physician and working in a public practice was protective against burnout, while a higher workload (more than 36 hours/week as on-call duties).

MBI - Maslach Burnout Inventory; EE - emotional exhaustion; PA - personal accomplishment; DP - depersonalization; ICU - intensive care unit.

## DISCUSSION

Burnout is particularly common in health-care professionals working in the emergency/critical care field, as shown by the Medscape physician lifestyle report in 2016,^(51)^ where the highest percentage of burnout occurred in critical care and emergency medicine physicians (55%), closely followed by anesthesiologists (50%).

Our systematic review aimed to summarize the findings on ICU physician burnout, since pooled data are currently available for all ICU personnel,^(30,31)^ but a summary on studies including data on burnout among ICU physicians only has not yet been conducted. During abstract screening, we noted that burnout in ICU staff was investigated more frequently in non-physician populations, with 85 studies being excluded because physicians were not involved. In our study, we undertook a significant effort to extrapolate data on physician burnout from studies including heterogeneous populations of critical care staff (i.e., including ICU nurses, nurse assistants, physiotherapists). Indeed, of the 17 included studies, only 5 focused on ICU physicians only, while the other 12 investigated physician burnout together with that of other critical care staff populations. Despite our efforts to enlarge the amount of data available by deep screening full texts, we were able to extract subgroup data regarding isolated physician burnout in almost half of the studies in mixed ICU populations (12 of the 26 selected initially). We also emailed the corresponding authors of the 14 mixed studies in order to expand the available data, but no one responded to our request. Nonetheless, the high heterogeneity already noted in the included studies suggests that the addition of further data would not have changed the main underlying message of our research: there is high methodological variability in studies investigating burnout in ICU physicians, and hazardous and meaningless conclusions from these studies should be avoided.

A previous systematic review on burnout in anesthesiologists found that different versions of the MBI questionnaire were used,^(52)^ thus hampering the interpretation of the results. For such reasons, we limited our appraisal regarding burnout among ICU physicians to studies using the MBI questionnaire, and we found that the vast majority used its full version. Despite this consistency, we found similar issues already brought up by the abovementioned systematic review on anesthesiologists:^(52)^ the included studies used very different cutoffs for EE, DP and PA. Moreover, we added an analysis on the Likert scale used for the MBI, and we found that this range varied greatly. Unfortunately, the cutoffs adopted for the diagnosis of burnout did not seem to correlate directly with such variability in the Likert-scale range (i.e., lower EE scores with a smaller Likert scale). Thus, any statistical or mathematical approach attempting to correct values or synthesize the reported levels of burnout in ICU physicians is meaningless. Of note, the included studies also gave different importance to the three domains. Some studies gave the same value to them, while others considered mainly EE and DP^(34,36)^ or EE only^(46)^ as pivotal domains in classifying high risk of burnout. In truth, a practical approach to easier interpretation of the MBI would be to get an overall result balancing the findings in the three domains, but such attempts to summarize the overall burnout levels were made only by a minority of studies (n = 4);^(41,42,44,49)^ even in these studies, the authors did not provide a clear explanation of the formula used to obtain the overall result, and different cutoffs were reported.

Some studies attempted to stratify the risk into low, moderate and high risk, while others defined only a high risk of burnout. We found the findings of a French survey very interesting; Garrouste-Orgeas et al.^(42)^ investigated burnout levels according to two different definitions, the first considering burnout as the presence of an alteration in all domains and the second evaluating the overall burnout score. The findings of the authors are striking in the sense that the first definition identified only 2.5% of physicians at high risk of burnout, while the second identified over 40%. In our belief, this finding again supports the idea that averaging literature findings provides biased conclusions; moreover, despite the attempt to reduce data heterogeneity by including only studies using the MBI questionnaire, our findings on the heterogeneous methodology of the included studies highlight the urgency for a consensus on burnout cutoffs when using the MBI questionnaire, together with clear reporting.

Apart from the difficulty of drawing conclusions, we found a variable response rate (from 30% to 90%). The response rate is a very important - and possibly underestimated - concept in the conduction of surveys because it may shift results on both sides. In the case of burnout, opposite interpretations are plausible. Indeed, it is possible that people at risk of burnout may not be keen on answering due to their disengagement in work-related issues and initiatives (such as a survey). Alternatively, it is possible that ICU physicians at risk of burnout show greater appreciation towards initiatives devoted to the support of workers, perceiving the importance of evaluating and addressing their work-related fatigue and sense of frustration.

### Limitations

Our systematic review has strengths and limitations. We performed a highly specific systematic review focusing on physician burnout in the ICU and included only studies using the MBI questionnaire, which is by far the most commonly used questionnaire to screen burnout. This decision was intentionally planned in order to - theoretically - obtain more comparable results. Although such an a priori decision was reasonable, the presence of several other weaknesses in reporting and methodological heterogeneities identified in our appraisal indicated that a numerical synthesis of the retrieved data was not warranted.

Importantly, we excluded studies in which the ICU personnel surveyed was not purely from the ICU but also consisted of surgeons and pediatricians working in the ICU. This approach permitted us to conduct a sectorial appraisal, but we still found high heterogeneity in the population of ICU physicians included. Indeed, several studies included ICU physicians at different stages of their careers (specialists and/or residents and/or interns) and variable ICU settings, ranging from any type of ICU to very specific ICU subtypes (in this regard, conducted mainly in the setting of pediatric and/or neonatal ICUs).^(39,46,50)^ We also identified single center studies as well as surveys conducted on regional to national (and one continental) scales. Another source of heterogeneity was related to the variability in response rate. To the best of our knowledge, there is no established cut-off for response rate to decide whether to include a study.

## CONCLUSION

This survey aimed to summarize data on the prevalence of burnout in intensive care unit physicians over the past 20 years. The appraisal of the published literature showed great heterogeneity in the methodological designs, including different scales for the evaluation and different cutoffs for burnout diagnosis. We believe it is urgent to achieve a consensus on methodological approaches for burnout evaluation.

**Take-home message:** Our systematic review on the prevalence of burnout in intensive care unit physicians, as evaluated by the Maslach Burnout Inventory questionnaire, found huge variability in the setting of the studies as well as in their methodologies, with variable definitions of burnout, different ranges used in the Maslach Burnout Inventory scale and mutable cut-offs. While it is impossible to draw conclusions on the true prevalence of physician burnout in the intensive care unit, it is urgent to establish a consensus on the methodology for conducting and reporting studies investigating burnout.
