# Erratum

**DOI:** 10.5935/0103-507X.20200102

**Published:** 2020

In the article **Prevalence of burnout among intensive care physicians: a systematic review**, with DOI number: 10.5935/0103-507X.20200076, published in the journal Revista Brasileira de Terapia Intensiva, 32(3):458-467, on page 458:

Where it read:

Mirko Minieri

Read:

Mirko Mineri

